# A commentary on ‘Effectiveness of local anesthetic application methods in postoperative pain control in laparoscopic cholecystectomies; a randomised controlled trial’

**DOI:** 10.1097/JS9.0000000000000744

**Published:** 2023-09-13

**Authors:** Zhichao Chen, Jianfeng Wei

**Affiliations:** Hepatological Surgery Department, Second Affiliated Hospital of Fujian Medical University, Quanzhou, Fujian Province, People’s Republic of China

*Dear Editor*,

It was with great interest that we read the article published in the *International Journal of Surgery* in 2022. The authors observed the effectiveness of local anesthetic injection (LAI), abdominal local anesthesia injection (IPLA), and transabdominal plane block (TAPB), and no local anesthetic controls on postoperative pain, and compared the analgesic efficacy of these methods^1^. The results showed that intraoperative local anesthesia can effectively control postoperative pain, especially TAPB can better manage postoperative pain. Many aspects of this study are well done. The authors conducted a double-blind, randomized controlled study. They had 160 patients who underwent laparoscopic cholecystectomy for cholecystitis and used statistical analysis to rule out confounding factors that might influence postoperative pain, such as age, gender, previous operations, body mass index and number of trocars. In addition, they openly discussed the limitations of their study. All of these are advantages of research design. We can learn from their examples. However, we believe that there are several important questions in this study that are not well addressed.

The authors mention in the ‘Materials and methods’ section, ‘All patients underwent laparoscopic cholecystectomy by two experienced surgeons by placing four trocars in the mid-abdomen and upper abdomen.’ However, we know that some of the steps and details of the operation may lead to different levels of postoperative pain. First, which port to retrieval the gallbladder from during the operation. Trauma to the abdominal wall during gallbladder removal is known to cause postoperative pain, and the gallbladder is usually removed through a puncture hole in the upper abdomen and umbilical cord. However, the location of removal can lead to different postoperative pain. Some studies have shown that gallbladder retrieval from the umbilical port with lower port site pain than gallbladder retrieval from the upper abdominal port in patients undergoing laparoscopic cholecystectomy^2^. Second, whether to place drainage tube during the operation. In clinical work, some gallbladder inflammation is severe, the closure of the gallbladder duct is not satisfied usually need to indentation drainage tube, to facilitate postoperative observation whether there is bleeding or bile leakage and other manifestations. The drainage tube is also an important factor that causes postoperative pain at the mouth of the drainage tube and mechanical stimulation^3^. Therefore, the location of gallbladder retrieval and the placement of drainage tube are likely to cause differences in postoperative VAS (Visual Analogue Scale) and satisfaction scores. However, this study did not report the relevant situation of these details during the operation.

In addition, the subjects of this study were patients undergoing laparoscopic cholecystectomy due to symptomatic cholelithiasis. In fact, chronic abdominal pain is very common in these patients and often causes psychological disorders such as anxiety. These psychological factors before surgery are also related to the degree of pain after surgery, and can affect patient satisfaction. Bayrak *et al*.^4^ reported that preoperative anxiety may cause intraoperative hemodynamic problems, increase patients’ need for postoperative analgesia, and reduce patients’ postoperative satisfaction. If the design of this study included preoperative assessment of patients’ anxiety, such as Spielberger’s State-Trait Anxiety Inventory (STAI) to use, the study would provide more useful data for effective pain control after laparoscopic cholecystectomy.

Despite these problems, this study provides a direction for addressing postoperative analgesia of the gallbladder. However, further research is needed to confirm the main findings of this study. Finally, we are very grateful to Ergin and his colleagues for their tremendous efforts in carrying out this very important study.

## Ethical approval

Not available.

## Consent

This is a commentary.

## Sources of funding

Not available.

## Author contribution

J.W.: manuscript preparation; Z.C.: critical revision and submission.

## Conflicts of interest disclosure

The authors Zhichao Chen and Jianfeng Wei declare that there are no conflicts of interest.

## Research registration unique identifying number (UIN)

This is a commentary.

## Guarantor

Jianfeng Wei.

## Data availability statement

This is a commentary; no data needs to be stated.

## Provenance and peer review

Not available.
